# Prime Editing Technology and Its Prospects for Future Applications in Plant Biology Research

**DOI:** 10.34133/2020/9350905

**Published:** 2020-06-26

**Authors:** Md. Mahmudul Hassan, Guoliang Yuan, Jin-Gui Chen, Gerald A. Tuskan, Xiaohan Yang

**Affiliations:** ^1^Biosciences Division, Oak Ridge National Laboratory, Oak Ridge TN 37831, USA; ^2^Center for Bioenergy Innovation, Oak Ridge National Laboratory, Oak Ridge, TN 37831, USA; ^3^Department of Genetics and Plant Breeding, Patuakhali Science and Technology University, Dumki, Patuakhali 8602, Bangladesh

## Abstract

Many applications in plant biology requires editing genomes accurately including correcting point mutations, incorporation of single-nucleotide polymorphisms (SNPs), and introduction of multinucleotide insertion/deletions (indels) into a predetermined position in the genome. These types of modifications are possible using existing genome-editing technologies such as the CRISPR-Cas systems, which require induction of double-stranded breaks in the target DNA site and the supply of a donor DNA molecule that contains the desired edit sequence. However, low frequency of homologous recombination in plants and difficulty of delivering the donor DNA molecules make this process extremely inefficient. Another kind of technology known as base editing can perform precise editing; however, only certain types of modifications can be obtained, e.g., C/G-to-T/A and A/T-to-G/C. Recently, a new type of genome-editing technology, referred to as “prime editing,” has been developed, which can achieve various types of editing such as any base-to-base conversion, including both transitions (C→T, G→A, A→G, and T→C) and transversion mutations (C→A, C→G, G→C, G→T, A→C, A→T, T→A, and T→G), as well as small indels without the requirement for inducing double-stranded break in the DNA. Because prime editing has wide flexibility to achieve different types of edits in the genome, it holds a great potential for developing superior crops for various purposes, such as increasing yield, providing resistance to various abiotic and biotic stresses, and improving quality of plant product. In this review, we describe the prime editing technology and discuss its limitations and potential applications in plant biology research.

## 1. Introduction

In the field of genome editing, there have been tremendous progresses over the past few years. However, an “all-in-one” perfect genome-editing technology, which can achieve any desired editing in the target DNA without any undesired effects, does not exist [1]. A major challenge of the existing genome-editing technologies is their inability to simultaneously introduce multiple types of edits such as small insertions/deletions (indels) and single-nucleotide substitutions in the target DNA sites [2–8]. A genome-editing technology that can perform these kinds of modifications will have tremendous potential for accelerating crop improvement and breeding [5, 9–13]. Precise genome-editing in plants can be achieved using CRISPR technologies via homologous recombination (HR) initiated by the induction of double-stranded break (DSB) at the target genomic site along with a donor DNA template that contains the desired edits [14–18]. However, the frequency of HR in plants is extremely low, and the delivery of the donor DNA to the target cell types is challenging [19–21]. An alternative to HR is the base-editing technology. However, current base-editing technologies can only perform substitution mutations, allowing for only four types of modifications (C/G-to-T/A and A/T-to-G/C), and they cannot instate insertions, deletions, or transversion types of substitution [22–24].

Anzalone et al. [25] recently developed a new genome-editing technique, called prime editing, that can overcome the aforementioned challenges. This new pioneering genome-editing technology can introduce indels and all 12 base-to-base conversions, with less unintended products at the targeted locus as well as fewer off-target events [1, 3, 25]. More recently, prime editing was applied to two plant species, rice [2, 26–29] and wheat [2], indicating that this technology holds tremendous potential for genome-editing applications in plants. Here we describe this technology, discuss important parameters affecting the editing efficiency, provide perspectives on how this technology might be improved to develop an “all-in-one” genome-editing technology for plants, and explore its potential applications in plant biology research.

## 2. The Principle of Prime Editing Technology

**Figure 1:**
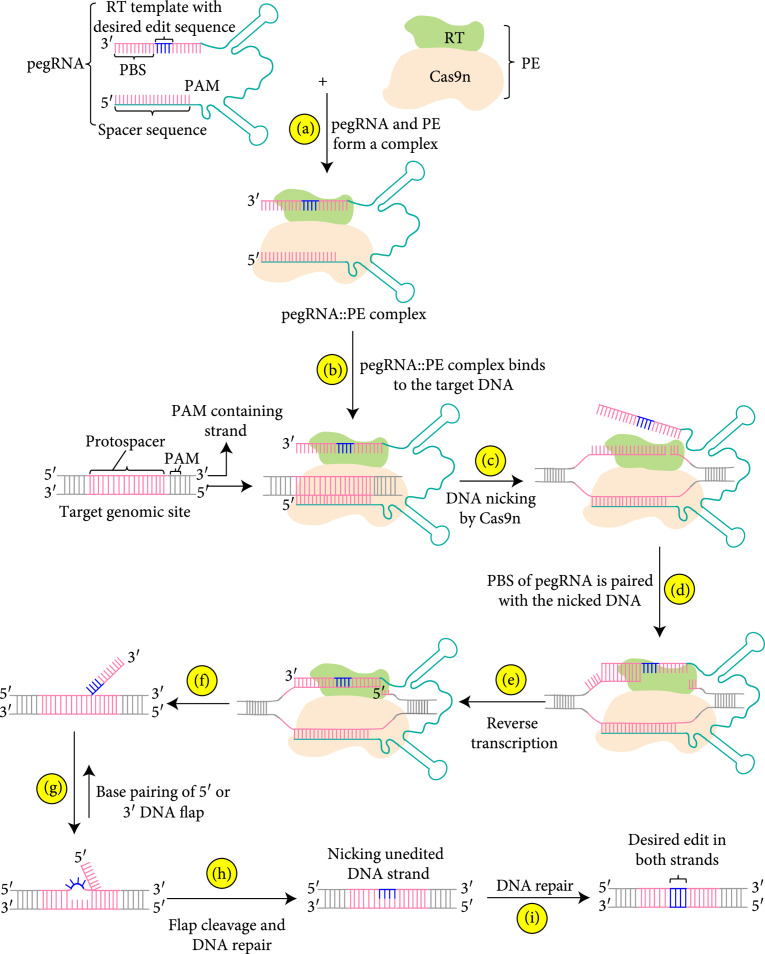
Schematic outline of principal events of prime editing technology. RT: reverse transcriptase; PBS: primer binding site; pegRNA: prime editing guide RNA; sgRNA: single-guide RNA; PE: prime editor; Cas9n: Cas9 nickase; PAM: protospacer adjacent motif.

The prime editing system is composed of two components: an engineered prime editing guide RNA (pegRNA) and a prime editor (PE) (Figure 1). pegRNA has a spacer sequence that is complementary to one strand of the DNA, a primer binding site (PBS) sequence (~8-16 nt), and a reverse transcriptase (RT) template that contains the desired editing sequence to be copied into the target site in the genome via reverse transcription (Figure 1). PE has a mutant Cas9 protein that can only cut one strand of DNA and is popularly known as Cas9 nickase (Cas9n) (Figure 1). The other component of PE is a RT enzyme that performs the required editing (Figure 1). Upon expression of a stably or transiently expressed prime editing construct, the PE and pegRNA form a complex (Figure 1(a)) that then moves to the target DNA site guided by pegRNA (Figure 1(b)). At the target site, Cas9n nicks one strand, which contains the PAM sequence, of the DNA, generating a flap (Figure 1(c)), and then the PBS of pegRNA binds to the nicked strand (Figure 1(d)). RT, an RNA-dependent DNA polymerase, is then used to elongate the nicked DNA strand by using the sequence information from the pegRNA, resulting in the incorporation of the desired edit in one strand of the DNA (Figure 1(e) and 1(f)). During this reaction, the nicked strand of the DNA binds to the PBS and acts as a primer to initiate the reverse transcription, leading to the incorporation of the desired edit from the RT template region to the PAM-containing strand. Following the completion of RT-mediated incorporation of the desired edit in the nicked DNA strand, the editing area contains two redundant single-stranded DNA flaps: an unedited 5${}^{\prime }$ DNA flaps (Figure 1(g)) and edited 3${}^{\prime }$ DNA flap (Figure 1(f)) [3, 25]. These single-stranded DNA flaps are eventually processed by the cellular DNA repair system and integrated into the genome. At the end of the editing, the nicked DNA strand is replaced by the edited strand through copying the sequence information from pegRNA, resulting in the formation of heteroduplex that contains one edited and one unedited strand (Figure 1(g)). A second nick is performed in the unmodified DNA strand using a standard guide RNA (Figure 1(h)) which is eventually repaired by copying the information from edited stand leading to the incorporation of desired edit in both strands of the DNA (Figure 1(i)).

## 3. Parameters Affecting the Efficiency of Prime Editing

Preliminary studies in plant and human systems have identified several factors that affect the efficiency of prime editing, including source of the RT enzyme, thermostability and binding capacity of the RT enzyme to its target site, length of the RT template, length of the PBS sequence, and position of nicking sgRNA in the unmodified strand [2, 25–27]. Among these factors, thermostability, length of the RT template, and its binding capacity to the target site showed significant effect on the editing efficiency in both plant and human cells [2, 3, 25]. A study in human and yeast cells showed that mutations (D200N, L603W, and T330P) in RT enhancing its activity at high temperature also increased the frequency of insertion and transversion-type of edits up to 6.8-fold compared to the nonmutated RT [25]. In addition, mutations that increase the thermostability of RT and its binding capacity to the target also improve the editing efficiency up to 3.0-fold [25]. Different RT from different sources also showed varying editing efficiency, as demonstrated by [2] that RT obtained from Cauliflower mosaic virus (CaMV) had lower editing efficiency than the Moloney murine leukemia virus (M-MLV). It was recently reported that RT template length had a strong effect on the editing efficiency, especially in plant cells, whereas editing efficiency was not improved significantly by changing the PBS length and position of nicking sgRNA [2, 27]. Secondary structure of pegRNA and G/C content of PBS region might also influence the editing efficiency [25]. Thus, a thorough testing of different kinds of pegRNAs and sgRNAs in combination with a wide range of target sites in various tissues or cells will be required to optimize the parameters for prime editing in plants.

Prime editing also has lower frequency of off-target effects than the conventional CRISPR-Cas9 genome-editing system [25, 26] . This low off-target activity has been attributed to prime editing involving three steps of hybridization between the spacer sequence and the target DNA, including hybridization between the target DNA and the spacer region of pegRNA, the PBS region of pegRNA, and the edited DNA flap [3]. In the traditional CRISPR-Cas gene editing system, only the hybridization between the target DNA and the protospacer from sgRNA is required for editing, which greatly increases the chances of off-target editing [30, 31].

Previous studies with base editors have found that induction of nick in the unmodified DNA strand increases the editing efficiency of base-editing system [22, 23, 32, 33]. A similar approach was also tested in prime editing, with improvement in editing efficiency only found in human and yeast cells, not in plant cells [2, 25, 27]. It was recently reported that editing efficiency might be influenced by temperature, with the editing efficiency (6.3%) at 37°C higher than that (3.9%) at a lower temperature (26°C), suggesting that the performance of prime editing system might be improved by testing alternate conditions and temperatures [2]. In addition, the sequence context of the target site might also highly influence the editing efficiency [2, 25]. In rice protoplast, editing efficiency was reported to be highly variable in different target sites of gene *OsCDC48*, with higher editing efficiency in the *OsCDC48-T1* site (8.2%) compare to the other two sites *OsCDC-T2* (2.0%) and *OsCDC48-T3* (~0.1%) [2]. Another recent study [27] revealed similar findings, where they found that the editing efficiency (1.55%) at the rice locus *OsDEP1* was higher than those (0.05-0.4%) at other loci (*OsALS*, *OsKO2*, *OsPDS*,*OsEPSPS*, *OsGRF4*, and *OsSPL14*). Other recent studies [28, 29] in plants also reported similar findings. In human cells, only the HEK293T line showed high editing efficiency (20-50%) whereas other lines tested showed relatively lower editing efficiency (15-30%) [25]. These data suggest that editing efficiency varies among target sites and different cell or tissue types, e.g., germline editing in *Arabidopsis*.

Types of mutations generated by the prime editing system can also be variable, with the frequency of certain kinds of mutation higher than the others, as demonstrated in recent reports [2, 26–29]. It was recently reported that the frequency of deletion (6 bp) could be up to 21.8% [27] and insertion (3 bp) up to 19.8% [29] whereas the frequency of point mutations ranged from 0.03% to 18.75% in rice [2, 26–29]. In wheat, the frequency of similar kinds of mutations was lower than that in rice, particularly the point mutation frequency, which was only 1.4% in comparison with 9.38% in rice [2]. In the case of all 12 base-to-base substitutions, the frequency of edits ranged from 0.2 to 8.0% [2]. In plant, it has been shown that frequency of indels decreases as the length of targeted insertion or deletion increases, with the longest inserted sequence and the longest deleted sequence being 15 nt and 40 nt in length, respectively [2]. Different prime editing systems in plants, their features, and editing efficiency are summarized in Table 1.

**Table 1:** Different types of plant prime editor (PPE), their features, and editing efficiency in different target sites.

Target gene	PPE type	PPE features (PBS^a^ length (nt), RT^b^ template length (nt), and RT type)	Mutation type	Editing efficiency^i^		Refs
				Desired	Undesired	
*BFP*	PPE3b	14, 12, M-MLV^c^	2 bp Subs^f^	4.40%	NR^j^	[2]
*BFP*	PPE3b	14, 12, CaMV^d^	2 bp Subs	3.70%	NR	[2]
*BFP*	PPE3b	14, 12, Retron^e^	2 bp Subs	2.40%	NR	[2]
*OsCDC48*	PPE2	12, 9, M-MLV	6 bp Del^g^	8.20%	4.6%	[2]
*OsCDC48*	PPE3	12, 9, M-MLV	6 bp Del	21.80%	NR	[2]
*OsCDC48*	PPE3	12, 15, M-MLV	3 bp Subs	2.60%	NR	[2]
*OsCDC48*	PPE3b	12, 9, M-MLV	6 bp Del	11%	NR	[2]
*OsCDC48*	PPE3b	12, 9, CaMV	6 bp Del	5.80%	NR	[2]
*OsCDC48*	PPE3	10, 17, M-MLV	1 bp Subs	14.30%	NR	[2]
*OsCDC48*	PPE2	12, 13, M-MLV	3 bp Ins^h^	1.98%	0.7%	[2]
*OsCDC48*	PPE2	12, 18, M-MLV	1 bp Subs	5.70%	3.8%	[2]
*OsCDC48*	PPE3b	12, 13, M-MLV	3 bp Ins	1.88	0.03%	[2]
*OsCDC48*	PPE3b	12, 18, M-MLV	1 bp Subs	3.0%	2.0%	[2]
*OsCDC48*	PPE3b	12, 18, CaMV	1 bp Subs	0.30	NR	[2]
*OsCDC48*	PPE2	12, 15, M-MLV	3 bp Del	~0.05%	~0.05%	[2]
*OsCDC48*	PPE3b	12, 15, M-MLV	3 bp Del	~0.05%	~0.05%	[2]
*OsALS*	PPE2	10-12, 16-17, M-MLV	1 bp Subs	0.28%	0.035%	[2]
*OsALS*	PPE3	12-13, 13-16, M-MLV	1 bp Subs	0.35%	0.52%	[2]
*OsDEP1*	PPE2	13, 13, M-MLV	1 bp Subs	0.10-0.3%	0.03-0.3%	[2]
*OsDEP1*	PPE3	13, 11-13, M-MLV	1 bp Subs	0.10-0.3%	0.1-0.2%	[2]
*OsEPSPS*	PPE2	13, 11-20, M-MLV	1 bp Subs	0.80-1%	0.3-0.6%	[2]
*OsEPSPS*	PPE3	13, 20, M-MLV	1 bp Subs	2.27%	2.66%	[2]
*OsEPSPS*	PPE3	13, 17, M-MLV	1 bp Subs	1.55%	1.53%	[2]
*OsEPSPS*	PPE2	13, 17, M-MLV	1 bp Subs	0.10	0.2	[2]
*OsEPSPS*	PPE3	13, 17, M-MLV	1 bp Subs	0.10	0.2	[2]
*OsLDMAR*	PPE2	12, 15, M-MLV	1 bp Subs	0.35%	0.1%	[2]
*OsLDMAR*	PPE3	12, 15, M-MLV	1 bp Subs	0.73%	0.1%	[2]
*OsGAPDH*	PPE2	12, 16, M-MLV	1 bp Subs	1.40%	0.16%	[2]
*OsGAPDH*	PPE3	12, 16, M-MLV	1 bp Subs	1.60%	0.24%	[2]
*OsAAT*	PPE2	12, 13, M-MLV	2 bp Subs	0.12%	NR	[2]
*OsAAT*	PPE2-R	12, 13, M-MLV	2 bp Subs	0.04%	NR	[2]
*OsAAT*	PPE3b	12, 13, M-MLV	2 bp Subs	0.20%	NR	[2]
*OsAAT*	PPE3b-R	12, 13, M-MLV	2 bp Subs	0.45%	NR	[2]
*TaUbi10-*	PPE2	13, 16, M-MLV	1 bp Subs	0.06%	0.13%	[2]
*TaUbi10*	PPE3	13, 16, M-MLV	1 bp Subs	0.20%	0.1%	[2]
*TaUbi10*	PPE2	12, 12, M-MLV	1 bp Subs	0.40-0.80%	0.1-0.2%	[2]
*TaGW2*	PPE2	11, 11, M-MLV	1 bp Subs	0.30%	0.03%	[2]
*TaGW2*	PPE3	11, 11, M-MLV	1 bp Subs	0.36%	0.12%	[2]
*TaGASR7*	PPE2	12, 18, M-MLV	1 bp Subs	1.40%	0.00%	[2]
*TaGASR7*	PPE3	12, 18, M-MLV	1 bp Subs	0.67%	0.00%	[2]
*TaLOX2*	PPE2	12, 14, M-MLV	1 bp Subs	0.30%	0.068%	[2]
*TaLOX2*	PPE3	12, 14, M-MLV	1 bp Subs	0.22%	0.05%	[2]
*TaMLO*	PPE2	12, 12, M-MLV	1 bp Subs	0.60%	0.00%	[2]
*TaMLO*	PPE3	12, 12, M-MLV	1 bp Subs	0.40%	0.00%	[2]
*TaDME1*	PPE2	13, 14, M-MLV	1 bp Subs	1.30%	0.07%	[2]
*TaDME1*	PPE3	13, 14, M-MLV	1 bp Subs	1.00%	1.0%	[2]
*HPTII*	PPE3-t	13, 28, M-MLV	3 bp Subs	9.38%	NR	[26]
*OsALS*	PPE2-WT	13, 13, M-MLV	1 bp Subs	0.05%	NR	[27]
*OsALS*	PPE2-V01	13, 13, M-MLV	1 bp Subs	0.10%	NR	[27]
*OsALS*	PPE3b-V01	13, 13, M-MLV	1 bp Subs	0.10%	NR	[27]
*OsKO2*	PPE2-V01	13, 19, M-MLV	1 bp Subs	0.13%	NR	[27]
*OsDEP1*	PPE2-WT	13, 13, M-MLV	1 bp Subs	0.01%	NR	[27]
*OsDEP1*	PPE2-V01	13, 13, M-MLV	1 bp Subs	0.15%	NR	[27]
*OsDEP1*	PPE3-V02	10, 22, M-MLV	1 bp Subs	0.03%	NR	[27]
*OsDEP1*	PPE3-V02	12, 19, M-MLV	1 bp Subs	0.23%	NR	[27]
*OsDEP1*	PPE3-V02	13, 13, M-MLV	1 bp Subs	0.67%	NR	[27]
*OsDEP1*	PPE3-V02	14, 17, M-MLV	1 bp Subs	0.35%	NR	[27]
*OsDEP1*	PPE3-V02	12, 11, M-MLV	3 bp Ins	0.90%	NR	[27]
*OsDEP1*	PPE3-V02	13, 17, M-MLV	3 bp Ins	0.50%	NR	[27]
*OsDEP1*	PPE3-V02	14, 25, M-MLV	3 bp Ins	0.075%	NR	[27]
*OsDEP1*	PPE3-V02	16, 14, M-MLV	3 bp Ins	1.53%	NR	[27]
*OsPDS*	PPE2-V01	13, 13, M-MLV	1 bp Subs	0.06%	NR	[27]
*OsPDS*	PPE3b-V02	10-16, 10-25, M-MLV	3 bp Ins	0.03-0.25%	NR	[27]
*OsPDS*	PPE2-V01	10-16, 10-25, M-MLV	3 bp Ins	0.05-0.86%	NR	[27]
*OsPDS*	PPE3-V02	10-16, 10-19, M-MLV	3 bp Ins	0.08-0.8%	NR	[27]
*OsEPSPS*	PPE3b-V01	13, 23, M-MLV	1 bp Subs	0.36%	NR	[27]
*OsEPSPS*	PPE3b-V01	13, 18, M-MLV	1 bp Subs	0.13%	NR	[27]
*OsGRF4*	PPE3b-V01	13, 15, M-MLV	1 bp Subs	0.16%	NR	[27]
*GFP, ALS, APO1*	Sp-PE2	13, 13, M-MLV	1 bp Subs	0-17.1%	NR	[28]
*OsSLR1*	Sp-PE3	13, 13, M-MLV	3 bp Del	0.00%	NR	[28]
*OsSPL14, APO2*	Sp-PE3	13, 13, M-MLV	24 bp Ins	0.00%	NR	[28]
*GFP, ALS, HPT*	Sa-PE3	13, 16-34, M-MLV	1 bp Subs	0-32.65%	NR	[28]
*OsPDS*	pPE2	13, 12, M-MLV	1 bp Ins	7.30%	NR	[29]
*OsPDS*	pPE2	13, 13, M-MLV	2 bp Ins	12.5%	NR	[29]
*OsPDS*	pPE2	13, 14, M-MLV	3 bp Ins	19.8%	NR	[29]
*OsPDS*	pPE2	13, 11, M-MLV	28 bp Del	0.00%	NR	[29]
*OsPDS*	pPE2	13, 11, M-MLV	1 bp Subs	0-31.25%	NR	[29]
*OsACC*	pPE2	10-15, 10-34, M-MLV	1 bp Subs	0-14.6%	NR	[29]
*OsACC*	pPE3	13, 10, M-MLV	1 bp Subs	10.4-18.75%	NR	[29]
*OsACC*	pPE3b	13, 10, M-MLV	1 bp Subs	6.25%	NR	[29]
*OsWX1*	pPE2	15, 31, M-MLV	1 bp Subs	7.30%	NR	[29]

^a^PBS: primer binding site; ^b^RT: reverse transcriptase; ^c^M-MLV: Moloney murine leukemia virus; ^d^CaMV: Cauliflower mosaic virus; ^e^Retron: retron-derived RT (RT-retron) from *E. coli* BL21; ^f^Subs: substitution; ^g^Del: deletion; ^h^Ins: insertion; ^i^Data obtained from the published graph using the WebPlotDigitizer software (https://apps.automeris.io/wpd/); ^j^NR: not reported.

## 4. Key Limitations of Current Prime Editing Technology in Plants

Even though prime editing is a major breakthrough in genome editing in plant, the technology is still in infancy, and further studies are thus required to realize its full potential. The first key limitation of PE is its low editing efficiency. It is well known that the editing efficiency of PE (0.03-21.8%) in plant cells is much lower than that (20-50%) in human cells [2, 25–27]. Until now, the prime editing system has only been tested on a limited number of target genes (twenty five target genes) in two monocot species (rice and wheat) in plants [2]. Therefore, there is a need to test PE in a broader array of plants including dicot species. Although considerable editing efficiency has been achieved in some target loci (e.g., 21.8% in OsCDC48-T1), lower editing efficiency (0.05-0.4%) was reported in other tested gene targets in plants [2, 26, 27]. Furthermore, wheat, a polyploid crop, showed low editing efficiency (1.4%) compared with rice, which is a diploid crop [2]. The second key limitation of prime editing is its short editing window (i.e., size of RT template length), with a standard size of 12-16 nt [25]). Although longer editing windows (30-40 nt) have been reported [2], the success of prime editing using long editing window depends on the sequence content of the target genomic region, with some target sites supporting long editing window whereas others not [2].

To overcome the aforementioned two key limitations of current prime editing technology, future studies need to focus on a deep understanding of the design principle of prime editing, optimization of parameters affecting the editing efficiency, and expansion of the editing window. Although there are some guidelines for designing prime editing systems for plant and animal cells [2, 25], the design principle of prime editing has not been studied comprehensively. The current recommendations are based on the experimental data from editing of a very limited number of genomic loci (twenty five endogenous loci in plants and 12 endogenous loci in human cells), including human cell lines, yeast cells, and the protoplast of rice and wheat [2, 25–27]. To gain a deep understanding of the design principle of prime editing and optimize the parameters affecting the editing efficiency, some important questions need to be addressed, including the following: [1] How stable are pegRNAs? [2] Does the chromosomal position of the target and sequence variability of the target sites affect the efficiency of editing? And [3] how does the PE system work? Answers to these questions will undoubtedly aid to design better versions of prime editor to increase editing efficiency and expand its capability of editing larger genomic regions.

Current prime editing system reported in plants can be used to modify only one target site at a time. However, many traits in plants are controlled by multiple genes or QTLs [34–38]. Also, activating a biosynthetic or metabolic pathway often requires editing multiple genes at the same time. Therefore, current prime editing system cannot be used to modify multiple genes simultaneously. Another technical limitation of prime editing is the size of prime editing construct (~20 kb) which is fairly large making it inefficient to transform into plant. The use of Cas9 orthologs that are smaller in size such as CasX [39] would reduce the size of the prime editor and facilitate the delivery of PE into plant cell.

## 5. Potential Applications of Prime Editing in Plant Biology Research

**Figure 2:**
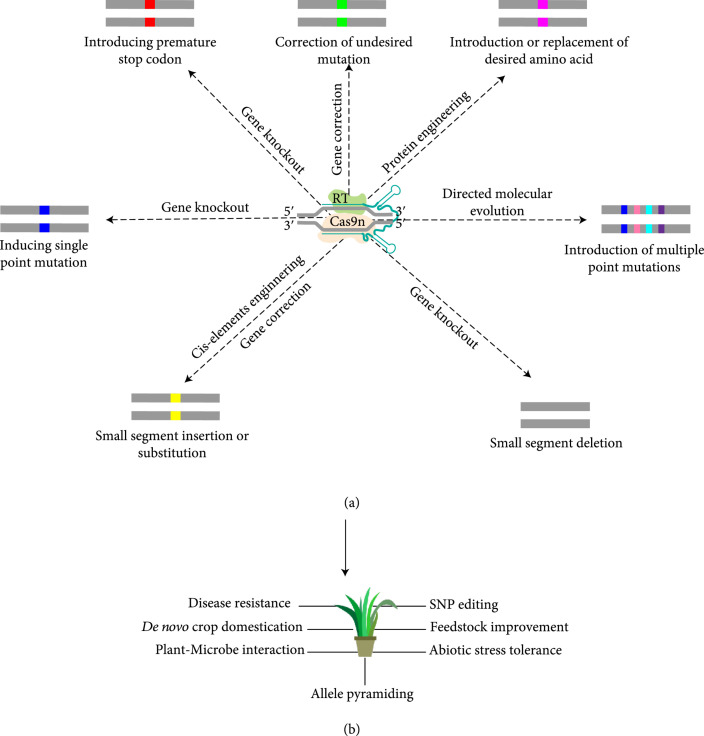
Possible genetic modifications mediated by prime editing and their potential applications in plant biology. (a) Different types of genetic modifications that can be potentially created using prime editing in plants. (b) Various applications of prime editing in plant biology research. Small rectangle indicates mutation, and different color within them denotes different mutations types. Medium-sized rectangle with yellow color indicates the segment of DNA inserted or replaced with prime editing. RT: reverse transcriptase; Cas9n: Cas9 nickase; SNP: single-nucleotide polymorphism.

The extraordinary ability of prime editing to generate targeted sequence modifications in genome has many potential applications in plant biology (Figure 2). This includes but is not limited to basic research, such as high-throughput analysis of gene function to improve annotation and generating artificial genetic diversity by directed evolution, as well as practical applications, such as engineering plants to improve yield, disease resistance, abiotic stress tolerance, and increase of the quantity and quality of useful chemicals in plants. Some of these applications are briefly described below.

### 5.1. Analysis and Editing of Gene Function through Prime Editing

Cellular processes in plants often involve genetic networks. Whole-genome sequences of many crops are publicly available, yet the function of most genes identified in genome sequence data remains unknown or hypothetical; thus, there is a need to apply gene editing technologies to improve gene annotation. Genetic manipulation of useful agronomic traits will require accurate annotation and precise engineering of complex biochemical or metabolic pathways. Therefore, a major goal of postgenomic era should be to systematically elucidate the function of all genes within subject organisms. Experimental characterization of the function of genes in plants will facilitate their deployment for various applications such as crop improvement and environmental sustainability.

Current genome-editing technologies such as CRISPR/Cas9 can efficiently generate loss-of-function mutants in plants [40, 41]; however, CRISPR/Cas9 have had limited success in gain-of-function studies (Table 2). CRISPR-activation (CRISPRa) can enhance the transcription rate of some genes, but this approach is not useful where a gene is nonfunctional due to the presence of premature stop codons or missense mutations. Base editing can be used to correct the premature stop codons or missense mutations; however, this approach has a limited flexibility, i.e., mostly involving transitions. As allowing for all 12 base-to-base substitutions, prime editing can create any base substitutions and thus help regain natural function of any mutated gene. In the model plant rice, it has been reported that nearly 65% SNPs are within the coding sequences [9]. Genome-wide association studies (GWAS) are continuously identifying new SNPs related to yield, disease resistance, salinity tolerance, drought tolerance, and many other important agronomic traits in a wide range of crop species [34–38]. Prime editing offers a great potential to verify the function of SNPs or indels predicted by GWAS.

**Table 2:** Comparison of prime editing with other gene editing technologies.

Areas of applications	Prime editing	Base editing	CRISPR-Cas9	TALENs	ZFNs
Generation of single point mutation	✓	✓	✓ (via HDR${}^{*}$)	✓ (via HDR${}^{*}$)	✓ (via HDR${}^{*}$)
Simultaneous introduction of multiple point mutations	✓	**×**	✓ (via HDR${}^{*}$)	✓ (via HDR${}^{*}$)	✓ (via HDR${}^{*}$)
Precise insertion	✓	**×**	✓ (via HDR${}^{*}$)	✓ (via HDR${}^{*}$)	✓ (via HDR${}^{*}$)
Precise deletions	✓	**×**	✓ (via HDR${}^{*}$)	✓ (via HDR${}^{*}$)	✓ (via HDR${}^{*}$)
Simultaneous introduction of insertion and deletions	✓	**×**	✓ (via HDR${}^{*}$)	✓ (via HDR${}^{*}$)	✓ (via HDR${}^{*}$)
Substitution (transition type)	✓	✓	✓ (via HDR${}^{*}$)	✓ (via HDR${}^{*}$)	✓ (via HDR${}^{*}$)
Substitution (transversions)	✓	**×**	✓ (via HDR${}^{*}$)	✓ (via HDR${}^{*}$)	✓ (via HDR${}^{*}$)
Directed gene evolution	✓	✓	✓	**×**	**×**
Generation of gene knockout	✓	✓	✓	✓	✓
Modification of cis elements	✓	**×**	✓ (via HDR${}^{*}$)	✓ (via HDR${}^{*}$)	✓ (via HDR${}^{*}$)
Gene activation^a^	✓	Limited scale	Limited scale	Limited scale	Limited scale
Multiplexing	Not tested yet	✓	✓	Limited scale	Limited scale

CRISPR: clustered regulatory interspaced short palindromic repeat; Cas: CRISPR associated; TALENs: transcription activator-like effector nucleases; ZFNS: zinc finger nucleases; HDR: homology directed repair. ^a^Gene activation: here, gene activation means the restoration of the activity of a gene that has mutation in the coding sequence. “${}^{*}$” indicates “extremely difficult or inefficient,” “✓” indicates “capable,” and “×” indicates “not capable”.

### 5.2. Generation of Artificial Genetic Diversity via Directed Evolution Mediated by Prime Editing

Directed evolution (DE), which is a process of making random mutation(s) in a target gene to artificially create genetic diversity [42], is another area where prime editor can play a key role. It is a powerful approach to improve performance of an existing gene or generate novel gene function and has been widely used for engineering novel enzymes, proteins, and antibodies with desired traits [43]. DE is usually implemented in prokaryotic systems such as bacteria or yeast [44]. However, a protein that is evolved in bacterial or yeast systems might not show the same function or behavior in other organisms such as plants and animals. It has been suggested that protein evolution experiment should be conducted in the target host [44]. However, technologies for DE have not yet been well established in higher eukaryotic hosts such as plants and animals. The CRISPR/Cas9 system is currently the prime genome-editing technology used for DE in eukaryotic organisms. CRISPR/Cas9-mediated DE uses a sgRNA library to introduce multiple random mutations in the target genes facilitated by Cas9-induced DSB induction to create a mutant population, which is then put under a selective pressure to evaluate the phenotype of the mutants harboring the evolved gene variants [44]. Unfortunately, most of the mutations generated by CRISPR/Cas9-mediated genome editing is likely due to frame-shift mutations, rather than in-frame, as the cellular DSB repair frequently results in the generation of small indels at the break sites. This is particularly an issue if the knockout mutants are inviable or not heritable making the mutagenesis power lost during selection. On the other hand, SNPs are the most common type of variations in different individuals of a single species, suggesting that generation of substitution mutations, particularly for making gain-of-function mutants, is more important than making indels for directed evolution [44]. Prime editing can thus be a very powerful approach for this purpose [45].

### 5.3. Genetic Improvement of Crop Plants Using Prime Editing

Various biotic and abiotic stresses, such as disease, salinity, drought, and heat, pose a serious challenge for crop production. They cause yield loss every year worldwide. Developing stress tolerant cultivars represents the most sustainable and eco-friendly way to alleviate these stresses. Prime editing can play a great role in developing new crops expressing stress tolerance. Due to the high precision of this technology, prime editing can be used to edit both coding and noncoding DNAs, providing new opportunities for precision crop breeding for increase tolerance to both abiotic and biotic stresses.

One of the promising applications of prime editing could be developing crops for disease resistance. Plant disease resistance genes are usually allelic in nature and vary only in single or a few nucleotides. It is known that because of the existence of missense mutations due to SNPs, certain alleles result in pseudogenes [46], leading to susceptibility due to loss-of-function. If the function of these pseudogenes can be recovered through prime editing, such genes might be able to impart disease resistance in crop plants. Alternatively, many crops resistant against nonviral pathogens are currently being engineered by genome editing through targeted mutagenesis of the so-called S genes, which negatively regulates defense [47]. Prime editing could provide a powerful approach for inactivating the S genes by introducing premature stop codons or nonsense mutations in their coding sequence. By exploiting the functional conservation of the S genes across different plant species, prime editing may be able to create desired S gene mutants of breeding value in most crop plants [46].

Prime editing technology could also be used to enrich repertoire of immune receptors that confer disease resistance. Immune receptors are plant proteins that regulate pathogen infection and activate cellular defense responses [48]. Prime editing may be used to accelerate the process of finding and validating new immune receptors in plant germplasms. Moreover, prime editing could be used to develop new variants of known immune receptors via directed evolution *in planta*. This will expand the arsenal of known immune receptors genes that may be deployed in the field. For example, the nucleotide-binding leucine-rich (NLR) family proteins comprise a large community of intracellular immune receptors that are found across plant and animal kingdom [49–52]. NLRs often detect the pathogen presence by binding to the pathogen-derived virulence factors and then mediating the modification of host target proteins [53]. Some of these host target proteins have evolved to function as virulence-targeted decoys [54]. Prime editing could be applied to each portion of the disease detection and signaling pathway to tune the resistance response. For example, in *Arabidopsis*, the NLR protein RESISTANCE TO PSEUDOMONAS SYRINGAE 5 (RPS5) activates the defense response [48]. However, this defense response depends on the activity of a decoy kinase protein PBS1 in the plant, which is cleaved upon binding to RPS5, resulting in the secretion of AvrPphB from *Pseudomonas syringae* into the plant cell [55]. It has been shown that by changing the cleavage sites of pathogen proteases, such as the AvrRpt2 protease from *Pseudomonas syringae* and the Nla protease from Turnip mosaic virus, in PBS1, the resistance spectrum of RPS5 could be expanded to other pathogens [56]. Similar kind of altered specificity and activity of immune receptors could also be generated via PE-mediated DE approaches *in planta*. Because prime editing can perform a wide range of mutations, this technique could be used to make multiple variants of useful immune receptor genes. Functional screening, in a synthetic biology context, can then be applied to identify gene variants conferring resistance phenotypes. This would broaden the application of directed molecular evolution for enhanced disease resistance in plants.

Besides improving plant resistance to pathogens, prime editing could be applied to the field of plant-microbe interactions among beneficial and/or symbiotic organisms, focusing on understanding the fundamentals of beneficial plant-microbe interactions in the context of sustainable farming to meet future food demands [57]. Previous works on beneficial plant-microbe interactions have typically focused on only a few model species [58]. In the recent years, extensive molecular studies on microbe-mediated plant benefits have been conducted to expand the applications of microbiome engineering for agriculture [59–63]. Prime editing might be a key technology in helping to understand the basics of plant-microbe interactions and to improve agricultural plants and microbes for beneficial use. Identifying individual plant or microbial candidate genes controlling beneficial traits could be facilitated using prime editing applications. However, essential questions that need to be addressed are, e.g., what molecular mechanisms are used by the rhizosphere microbiota to influence plant responses? Which genes in plants help shape the microbiota in rhizosphere? And, how do microbes and plants communicate with each other? Addressing these questions and others with prime editing would establish a direct link between agronomic traits and plant or microbial genes, accelerating the design of artificial microbial communities for improving crop productivity [57]. For example, one promising applications of prime editing could be decoding the role of effector molecules in plant and microbes which are involved in symbiosis. Genome-wide analysis have identified many effector molecules such as small secreted proteins (SSPs), which may play decisive role in symbiosis between *Laccaria bicolor* and *Populus trichocarpa* [64, 65]. While most of these SSPs are secreted by *L. bicolor*, a few of them [15] were found specific to *P. trichocarpa* [64]. Although the function of some of the SSPs secreted by *L. bicolor* has been decoded [66–69], the role of most of the SSPs in symbiosis is yet to be determined. Particularly, the role of plant-secreted SSPs in mediating symbiosis between the *L. bicolor* and *P. trichocarpa* is currently unknown. Prime editing could be used to generate loss-of-function phenotype to investigate the role of poplar (*Populus* spp.) secreted SSPs during symbiosis with *L. bicolor*. If any of the plant SSPs have an effect on the regulation of poplar-*Laccaria* symbiosis, prime editing could be used to engineer a novel version of the SSPs to improve the interaction between the bioenergy crop poplar and the mutualistic fungi *L. bicolor* [70–72].

Beyond biotic stress tolerance, prime editing also could be used to generate crop plants for tolerance to abiotic stresses, such as salinity, drought, and heat stress. As prime editing can precisely generate all types of base conversion and control small indels, this technology is ideal for editing cis-regulatory elements (CREs) to create novel trait variants. CREs are noncoding DNA regions known as promoters and enhancers [73], which regulate transcription of genes [74], and they contain binding sites for different transcription factors (TFs) or other regulatory proteins that can affect transcription [75]. [76, 77] have shown that mutations in the CREs can alter gene expression level and speed up the evolutionary process to domesticate crops via reshaping the landscape of transcriptome. Moreover, [78] found that almost half of the mutations responsible for crop domestication are in the CREs. Finally, a recent study [79] revealed that the number of CRE mutations associated with the crop domestication was even more than that was previously estimated by [78]. CREs are mostly found in the promoter region of genes, and their presence, absence, or variation of position in the promoter region regulates the gene expression and could induce, reduce, or turn-off gene expression [80]. For example, putative TFs *OsERF922* and *GhWRKY17* bind to the CRE sequence GCC box (AGCCGCC) and W-box (TTGACC), respectively, resulting in the susceptibility to abiotic stress tolerance such as drought and salinity [81, 82]. If the binding site of these putative TFs in the GCC-box and W-box could be altered, it might be possible to generate novel drought and salinity tolerant crops. Precise single-base mutations or indels within the W-box or GCC-box could abolish the binding site of putative TFs and might result in improved tolerance to drought and salinity. In *Arabidopsis thaliana*, several genes (*GST*, *P5CS*, and *POD SOD*), which are involved in stress response, are found to be negatively regulated by a TF *ANA069*, which interacts with the CREs of these genes and specifically binds to the sequence C[A/G]CG[T/G]; and when the core binding sequence in the CRE was mutated, plants showed enhanced abiotic stress tolerance [83]. By making random variations in the promoter regions with prime editing, it might be possible to generate novel phenotype and new QTLs for various traits like heat or drought tolerance. In fact, one study [84] showed that mutations in the promoter region could create a spectrum of phenotypic variations and generate unique QTLs for improved fruit size and yield in tomato. It has been previously reported that complete loss- or gain-of-gene function frequently showed deleterious pleiotropic effects [78]. On the other hand, a fine-tune gene expression without any pleiotropic effects may be achieved by inducing targeted mutation in the CREs. Therefore, precision engineering of cis-regulatory elements via prime editing represents a new tool in crop breeding.

Finally, prime editing could be applied to the development of and accelerate the domestication of emerging crops and plant-based feedstocks within the incipient bioeconomy. For example, prime editing could be used to modify or engineer genes involved in cellulose and hemicellulose biosynthesis and thereby increasing polysaccharide content of cell wall. Even though cellulose biosynthesis in plants has been studied for a long time, the complete molecular basis of cell wall biosynthesis is still poorly understood [85–97]. For instance, even in the model plants such as *A. thaliana*, most of the enzymes involved in cellulose biosynthesis have been identified based on hypothetical modelling, and their actual role in the cellulose synthesis pathways remains unknown [98–103]. Our current understanding of hemicellulose biosynthesis is even less comprehensive [104–106]. Future studies, using prime editing, could focus on understanding the biosynthesis of plant cell-wall polysaccharides, and their genetic manipulation, to increase polysaccharide feedstocks in the development of cellulosic-based biofuels and bioproducts. In a recent study, [86] established that cellulose biosynthesis in *Arabidopsis* was negatively affected by the *FLAVIN-BINDING KELCH REPEAT*, *F-BOX 1* (*FKF1*) gene, suggesting that cellulose production can be improved by inactivating the function of the *FKF1* gene. Knocking out or inactivating the function of a gene in plants with the conventional genome-editing technologies such as the CRISPR/Cas9 system requires the creation of DSB in the genome that might have deleterious effects on plant survival, and thus not suitable for precise engineering plant genome. Alternatively, prime editing offers higher precision and accuracy compared with a CRISPR/Cas9 system. Another classic example where a conventional CRISPR/Cas9 system is unable to produce the desired edits is the *Populus tomentosa CELLULOSE SYNTHASE GENE* (*PtoCesA4*) gene, which is directly related to cellulose biosynthesis and contains two SNPs (i.e., SNP-18 (T/A) and SNP-49 (C/A)) abolishing its function [107]. Correcting T/A or C/A mutation is not possible with CRISPR/Cas9-mediated genome-editing or base-editing system whereas prime editing offers the promise of introducing such mutations in an efficient manner, as shown by [*2*].

## 6. Conclusion and Future Perspectives

To achieve a variety of editing applications with a single technology, in a living system at highest resolution level, has been a major challenge until the advent of prime editing. With the tremendous potential of prime editing in precise genome editing, we are likely to witness rapid progress in a creative use of this new technology in plant biology research in the next few years. However, many challenges, such as low efficiency, limited editing window, unknown cell, tissue, and species-specificity, need to be overcome to realize prime editing’s full potential for applications in plant biology.

Prime editing in plants has low efficiency compared to human cells. The low efficiency of prime editing might be related to the expression level of pegRNA in plants. All the studies so far reporting prime editing in plant used a RNA Pol III promoter such as the U6 promoter to express the pegRNA. Previous studies have shown that RNA Pol II promoter such as the Cestrum yellow leaf curling virus (CmYLcv) promoter can improve the editing efficiency of CRISPR/Cas9 system up to 2-folds in plants. Therefore, one way of improving editing efficiency might be the use of alternative RNA polymerase promoter such as CmYLcv or U3 to express pegRNA. One of the major limitations of prime editing is its short editing window (12-16 nt) which limits its flexibility to insert or delete large DNA segments from the targeted genome. Thus, one of the major foci of future improvement in prime editing technology would be to investigate how to improve the editing window. Particularly, it needs to be investigated why some targets support long editing windows and others do not.

In addition to addressing the two key limitations (i.e., low editing efficiency and short editing window) mentioned above, future research should also investigate when the system does not work as expected. Off-target editing, including undesired effects on the genome, represents a major challenge in the previous genome-editing technologies such as the CRISPR/Cas9 system. Although prime editing has lower off-target activities than other genome-editing technologies [25], future work is still needed to further minimize the side effects of the prime editing technology in plants through a meticulous analysis of undesired effects of editing in the genome, including genome-scale investigation of off-target editing as well as a strong understanding of cellular impact.

Although prime editing has tremendous flexibility to achieve different types of mutations, it still requires the presence of specific PAM sequence in the target site which poses a difficulty in targeting any chosen site in the genome. The discovery of new class of Cas9 protein that has more plasticity to PAM requirement would broaden the scope of targeting site in the genome. A recent study [108] reported an engineered Cas9 protein that can target nearly any site in the genome without specific PAM requirement. Similar engineered Cas9 proteins could be tested in prime editing to broaden the scope targeting region in the genome. In addition, a previous gene editing system such as CRISPR/Cas9 can be multiplexed to edit several loci at the same time in plant. However, it is unknown whether a similar approach would work in plants for prime editing. One of the future improvements should therefore focus on the development of multiplexed prime editing system for plants to allow editing multiple loci at the same time. To achieve editing at multiple-target loci at the same time, several pegRNAs may be combined in a single polycistronic transcript using the endogenous tRNA processing system as shown in *Arabidopsis* for CRISPR/Cas9 system [109].

Prime editing technology is early phase of its development. It has some technical limitations and needs more research to optimize the system for plant. Here we have highlighted some key limitations of the system and provide some suggestion on how to improve it further. Despite some technical limitations and challenges, it is evident that prime editing will play a leading role among the many genome-editing technologies for basic plant biology research and crop improvement in near future.

## Data Availability

Submission of a manuscript to *BioDesign Research* implies that the data is freely available upon request or has deposited to an open database, like NCBI. If data are in an archive, include the accession number or a placeholder for it. Also include any materials that must be obtained through an MTA.
